# Contrasting Selective Pressures on Seed Traits of Two Congeneric Species by Their Main Native Guilds of Dispersers on Islands

**DOI:** 10.1371/journal.pone.0063266

**Published:** 2013-05-07

**Authors:** Manuel Nogales, Aarón González-Castro, Patricia Marrero, Elsa Bonnaud, Anna Traveset

**Affiliations:** 1 Island Ecology and Evolution Research Group (Instituto de Productos Naturales y Agrobiología–Consejo Superior de Investigaciones Científicas), La Laguna, Canary Islands, Spain; 2 School of Biological Sciences, University of East Anglia, Norwich, England; 3 Unité Ecologie, Systématique et Evolution, Equipe Ecologie des Populations et des Communautés, Université Paris Sud, Paris, France; 4 Laboratorio Internacional de Cambio Global, Institut Meditarrani d’Estudis Avançats (Consejo Superior de Investigaciones Científicas–Universitat de Les Illes Balears), Esporles, Balearic Islands, Spain; University of Marburg, Germany

## Abstract

Many fleshy-fruited plants from the Mediterranean and Macaronesian islands are dispersed through endozoochory. In mainland Mediterranean areas, reciprocal adaptations have been found between plants and animals, although evidence is scarce. On small isolated oceanic islands, such reciprocal adaptations might well be more prevalent due to intrinsic island traits. Here we evaluate the existence of selective pressures exerted by two different disperser guilds (lizards and birds) on two seed traits (seed coat thickness and seed germination pattern) of two congeneric species present on Mediterranean and Macaronesian islands. In the continental Balearic Islands, *Rubia peregrina* has evolved mostly with birds, although frugivorous lizards are present in some of these islands and are known to eventually consume its fruits. By contrast, *R. fruticosa,* endemic to the Macaronesian archipelago, has evolved mostly interacting with lizards and only recently with birds. We hypothesized that *R. fruticosa* would be especially adapted to saurochory, with thicker seed coats and higher germination proportion, whereas *R. peregrina* would be more adapted to ornithocory, with thinner seed coats and showing a lower germination percentage after being ingested by lizards. Captivity experiments of seed ingestions by natural and non-natural dispersers (i.e., frugivores that have not evolved with those plants) were conducted. Results suggest that dispersers did not exert any strong enough selective pressure to induce changes in germination patterns. We attribute this to the fact that the Rubiaceae is an ancestral family in the Mediterranean (both on continent and islands) and thus probably interacted with lizards in the past. Lastly, although we hold that the seed coat structure of *R. fruticosa* is probably associated with its evolutionary success after a long interaction with insular lizards, our findings support the idea that the relationship between endozoochorous plants and the guild of dispersers with whom they evolved is rather unspecific.

## Introduction

Most seed dispersal studies examine how vertebrates interact with fleshy-fruited plants from an ecological viewpoint and only rarely from an evolutionary perspective. Although botanists have been interested in the natural history of seed dispersal for many years [1], [2], attention from evolutionary ecologists has grown since the 1970s. The important theoretical approaches by some authors [3]–[7] inspired other scientists and brought further advances. A large number of these studies were performed both in tropical forests [8], [9] and in the Mediterranean Region (see reviews in [10], [11]), focusing mainly on different frugivore and fruit traits. In the former case, factors such as migratory behavior, seasonal feeding preferences, digestive physiology and tolerance to secondary compounds in fruit pulp were suggested to have ‘coevolved’ with frugivorous behavior. Fruit traits, by contrast, appear to mainly reflect the influence of phylogenetic correlations and historical contingencies [10], [12]. In the Mediterranean, the deep-rooted phylogenetic components that explain many fruit traits might actually be related to the fact that dispersers have not exerted a strong selective pressure on them. Plants are known to be conservative in a large number of traits, including fruit and seed characteristics [12]–[14].

There are several reasons for which coevolution between fleshy-fruited plants and frugivores are unlikely. One is the very limited ability of plants to direct seed dispersal towards safe sites for seedling emergence and survival. Another is the fact that selective pressures by animals can be diluted by environmental factors (both biotic and abiotic) before and after the dispersal process (e.g. [6], [15], [16]). Furthermore, long-term studies have indicated that interaction patterns between plants and dispersers are highly variable between years [17] and that abiotic factors are more determinant on fruit animal-interactions than biotic ones [18].

Especially in mainland ecosystems, interactions among members of a plant-frugivore assemblage are often complex, with a large number of frugivorous species (see [8], [9], [19], [20] and references therein). This constrains reciprocal adaptation between pairs of mutualist species [21]. However, such adaptations by specialized mutualists are feasible in isolated ecosystems [6], [16], where dispersers can play an important role in the evolution of plant traits. Curiously, the few studies that report a strong dependence of plants on animals have been all carried out in island ecosystems (e.g. [22]–[26]).

Almost all the above mentioned studies about fleshy-fruited plant evolution are focused on the characteristics of the fruits concerned (color, size, smell, chemical compounds, phenology, etc.). However, other traits related with seeds, and their interaction with the dispersers (e.g., seed size, coat thickness, viability, potential for germination) has received much less attention from an evolutionary perspective. This is so despite some of these seed traits are altered after ingestion by frugivores, with consequences for final germination and seedling growth [27], [28], and hence for disperser effectiveness (*sensu* [29]). In this study, we evaluate for the first time if two guilds of dispersers (lizards and birds) under isolation conditions can exert differential selective pressures on seed traits (seed coat thickness and potential for germination) of two related congeneric plant species from the Mediterranean Region: *Rubia peregrina* from the Mediterranean basin (Balearic Islands) and, presumably its nearest relative, *R. fruticosa* which is endemic to the central archipelagos of the Macaronesian islands (Madeira, Salvages and the Canaries).

*R. peregrina* fruits in the Mediterranean (including the Balearic archipelago) are currently consumed by a wide variety of both sedentary and migratory passerine birds, especially warblers ([30], [31], A. Traveset, pers. obs.). The lizards *Podarcis* spp. endemic to the Balearic Islands are also frugivores [32] interacting with *R. peregrina* [33]. However, this interaction is rarer as the fruiting period of *R. peregrina* is mainly in autumn, a season when lizards (exothermic) are inactive most of the time. Moreover, the *Podarcis* body size is smaller (snout-vent length of *P. lilfordi*: 70–80 mm) than the Canary Island endemic lizards *Gallotia* spp. (snout-vent length of *G. galloti*: 107–145 mm), which leads to different gut passage times. The effect of gut treatment on seeds would thus not be comparable between these species. Unlike mainland populations, *R. peregrina* in the Balearics has only interacted with mammals (specifically) after their introduction by humans 5000 years ago [34]. By contrast, *R. fruticosa* fruits in the Canaries are especially consumed by the large, highly abundant *Gallotia* lizards (in some cases almost 20 times more abundant than passerine birds, A. González-Castro, in prep.), especially in arid environments, although they are also consumed by birds [35]–[37]. In addition, seed dispersal and seedling recruitment of *R. fruticosa* depends more on lizards than birds (A. González-Castro, in prep.). Moreover, the genus *Gallotia* has been on the Canary Islands for 17–20 million of years [38], whereas the arrival of the passerine birds that currently inhabit the Canaries was approximately 4–13 thousand years ago [39] (see also review in [40]). Therefore, the interaction between *R. fruticosa* and *Gallotia* spp. seems older than that between this plant and birds. There is a wide difference in gut treatment between these two types of dispersers; seeds eaten by frugivorous lizards are subject to harsher processing as they pass through the entire digestive tract and are retained for a clearly longer time than the brief digestive treatment by frugivorous birds, e.g. warblers [41], [42]. The interaction between *Rubia* spp. and their seed dispersers in these island habitats therefore seems to provide an ideal system to test for seed adaptations to different disperser guilds.

In particular, we hypothesized that seeds of *R. peregrina* from the Balearics are adapted to the passage through the intestines of birds and would thus show a lower survival and coat resistance to lizard gut treatment. Considering that the abrasive effect on the *Rubia* seed coat is greater in lizard than in bird guts [43], we predict that (1) seed coats are thicker in *R. fruticosa* than in *R. peregrina*, and (2) the potential for germination of *R. peregrina* seeds is reduced to some extent when they are ingested by non-natural frugivorous lizards.

## Materials and Methods

### Ethics Statement

All necessary permits were obtained for the described field study. To perform fieldwork in Tenerife Island (The Canaries) we accounted with permission provided by the landowner of the study area, Teobaldo Méndez. In the case of Mallorca (Balearics), the Finca Pública de Son Real allowed us to perform fieldwork in its lands. Bird and lizard species studied in this work are listed in the UICN Red List as "Least Concern", thus special management methods were not required. Specific permission was not required either to collect *Rubia* fruits. The blackcap used in experiments was born in captivity, whereas lizards were caught by mean of pitfall live-traps. The ethical committee of Cabildo Insular de Tenerife granted the permit (no. 81512) to capture these lizards and to perform the captivity experiments according to the World Medical Assembly’s Declaration of Helsinski of 1964 on human and animal rights, as well as the guidelines proposed by the Association for the Study of Animal Behaviour [44]. For our objectives, it was not necessary to sacrifice any of the individuals. Moreover, none of the animals suffered any damage during experiments. According to animal welfare, lizards were kept separately in individual terrarium and the blackcap in a wide birdcage. Lizards were fed with a mixture diet based on tomatoes, *Bituminaria bituminosa* leaves, *Tenebrio molitor* larvae and water. The blackcap was fed with its usual mixed diet of feed, *Tenebrio molitor* larvae and water.

### Study Areas and the Two *Rubia* Species

The Balearic Islands are four main islands of continental origin in the Mediterranean Sea, located between 40°–38°N and 4°–1°E, 75 km off the eastern Iberian peninsula and 380 km from the north coast of Africa. Seeds of *R. peregrina* used in this study were collected during November 2009 in Son Real (in the NE of the largest island, Mallorca) at about 35 m a.s.l., where the climate is typically coastal Mediterranean (annual rainfall: ≈600 mm per year, and mean temperature around 17°C). On the other hand, the Canaries are seven main islands of volcanic origin situated between 27°N–29°N and 13°W–18°W, less than 100 km off the Atlantic coast of north-west Africa. Seed material of *R. fruticosa* was collected during March 2010 in Teno (NW corner of Tenerife) about 200 m a.s.l., where the climate is semiarid (annual rainfall: ≈200 mm per year, and mean temperature around 20°C).

The genus *Rubia* (Rubiaceae) includes approximately 60 species of perennials widely distributed over Europe, Africa, Asia and America. *Rubia peregrina* and *R. fruticosa* are inhabitants of the Mediterranean Region. *Rubia peregrina* is widely distributed around the Mediterranean basin and Macaronesia (Azores, Madeira and Canaries). However, in the Canaries, this species is restricted to moist laurel forests, where the role of lizards as dispersers is insignificant. *Rubia peregrina* exhibits a climbing herb biotype that can reach more than 4 m in height, and it is widespread in the Balearics. Fleshy fruits are black spherical berries (color typically associated with consumption by birds [45], [46]). In fact, the dispersal syndrome of this species has recently been regarded as ornithochorous [47]. These fruits are 4.9 mm in length and 4.4 mm in diameter, with 1.3 seeds per fruit [33], and are present from October to January. In the Canaries, *R. peregrina* is located at higher altitudes (≈400–1200 m a.s.l.), where lizards are not present (or scarce) and thus birds exclusively disperse the plant. On the other hand, *Rubia fruticosa* shows a sub-shrub biotype that can reach 0.49 m^2^ of plant cover and 0.54 m in height [43]. It is present on all the seven main Canary Islands and occupies the coastal xerophytic scrub, although it can also reach high altitudes (≈800 m a.s.l.) when the original vegetation belts have been profoundly modified by humans [48]. Its fleshy fruits are spherical translucent berries (length: 7.5 mm; diameter: 5.6 mm; no. of seeds per fruit: 1.4), present from early March until May [49]. This species is dispersed by several native vertebrates, including the endemic lizards *Gallotia* spp. ([35], [43] and references therein), small (warblers), medium (blackbirds) and large passerines (ravens) [43], [49], and omnivorous non-passerine birds (e.g. gulls, [50]).

### Experimental Procedures

A total of 200 fruits per mother plant (*n* = 20 randomly selected mother plants per species) were collected on the two islands and stored at room temperature until frugivore experiments were performed. In order to detect any possible mother-plant effect we considered the individuals from which seeds were collected throughout the experiment.

A total of 40 adult *Gallotia galloti* lizards were kept in short-term captivity (≈2 weeks) and subsequently released where they were captured. During this captivity period, they were additionally fed with a mixed diet of plant and animal matter (leaves of the Fabaceae *Bituminaria bituminosa* and commercial Coleoptera larvae of *Tenebrio molitor*). Half of the lizards (20) were fed with 800 *R. peregrina* fruits (40 each) while the other half received the same amount of *R. fruticosa* fruits. Simultaneously, due to problems with adaptation of adult Canarian blackcaps *Sylvia atricapilla* to captivity, we used an individual born in captivity to obtain avian-ingested *Rubia* seeds, which greatly facilitated this task. When comparing the germination ratio of *Rubia fruticosa* seeds defecated by the captive individual with that of *R. fruticosa* seeds from the same study area (Los Adernos) defecated by wild warblers (Sylviidae), no significant difference was found (*G_1_* = 3,355, *P* = 0.07). Therefore, there is no evidence that the gut treatment provided by the captive experimental bird is not representative of that by *Sylvia* spp. under natural conditions. This individual was fed with *Rubia* spp. fruits and we obtained more than 1600 *Rubia* seeds, the same number that seeds ingested by lizards (800 from *R. peregrina* fruits and 800 from *R. fruticosa* fruits; 40 seeds per mother plant). All these experiments in captivity were performed during spring-summer 2010 and seeds defecated by both groups of vertebrates were conserved at room temperature until the seedling emergence experiment started. Control seeds were obtained by fruits collected in the same mother plants as those used to feed the dispersers. We obtained a total of 5200 seeds (*n* = 1600 seeds per disperser treatment: lizards and birds, and 1000 control seeds for each species of *Rubia*).

Coat thickness was measured for each species and treatment (lizards, birds and control). Five seeds per mother-plant were measured (*n* = 10 repeated measurements per seed) for each treatment, using an AxioCam MRc5 (Zeiss) on the stereo-microscope Zeiss Stemi SV 6 with x5 ocular magnification plus the Axiovision software package (version 4.7.1.0).

The experiment on seedling emergence for all treatments and plant species was carried out in a greenhouse over a period of 6 months (15 October 2010–5 April 2011). A total of 1600 seeds from each *Rubia* species were planted. For each of the 40 mother plants (20 per species), we sowed: 40 controls (20 with pulp and 20 depulped, following the recommendations of [51]; 20 seeds ingested by *G. galloti*, and another set of 20 seeds, ingested by *S.* *atricapilla*. Each individual seed was sown 5 mm deep independently in a 27 cm^3^ pot (*n* = 104 pots per tray) containing a standard substrate (25% turf, 25% river sand, and 50% agricultural soil). Plots were watered every two days and seedling emergence was monitored every five days. Seeding emergence (hereafter ‘seed germination’) was noted when a seedling appeared above the soil surface. All experiments of this study were carried out at Tagoro (UTM 28RCS3623154) in northeastern Tenerife at 300 m a.s.l.

### Statistical Analyses

Differences in seed coat thickness among the three treatments (control, lizards and birds) and between the two species (*R. peregrina* and *R. fruticosa*) were tested by means of a Generalized Linear Model (GLM), with the response variable (seed coat thickness) log-transformed. When a significant effect due to treatment was found, pairwise comparisons between treatments were performed with new significance levels calculated by mean of the Bonferroni correction factors (α’ = 0.016).

To test for the effect of treatment, species and mother plant on seed germination ratio we applied a mixed GLM fitted to a binomial error distribution, with the effect of each mother plant as a random effect factor. In this case, for pairwise comparisons between treatments we also established a new significance level by the Bonferroni correction factors (α’ = 0.0018). In addition, we applied Spearman’s correlation test to assess if there was a relationship between average seed coat thickness and seed germination ratio of each mother plant, within each treatment. Survival analyses of seed germination from mother plants of both species were carried out by applying the Kaplan-Meier analysis (Breslow tests). It allowed us to test for differences in seed germination rate between treatments and plant species. Statistical analyses were performed using the SPSS statistical package (version 18.0) and the “base” package implemented in R 2.11 [52].

## Results

### Seed Coat Thickness

Seeds defecated by either lizards or birds appeared to be intact, with no external visual damage. Seed coats of *R. peregrina* were much thinner than those of *R. fruticosa* (*χ* ^2^ = 225.73, *df* = 1, *P*<0.001; Table 1). In both *Rubia* species the two dispersers tended to decrease seed coat thickness, especially lizards (Table 1). The effect of digestive treatment was not significant in *R. peregrina* (*χ* ^2^ = 4.16, *df.* = 2, *P* = 0.125) but it was in *R. fruticosa* (*χ* ^2^ = 7.39, *df* = 2, *P* = 0.025; Table 1). In the latter species, lizards significantly reduced the seed coat as compared to control seeds, whereas the effect due to bird treatment was intermediate (Table 1).

**Table 1:** *Rubia peregrina* and *R. fruticosa* seed coat thickness (µm).

	*R. peregrina*			*R. fruticosa*		
	Mean	SD	*n*	Mean	SD	*n*
Control	24.56^a^	7.41	100	66.66^a^	28.04	100
Lizards	22.79^a^	5.03	94	57.60^b^	28.87	93
Birds	23.34^a^	5.82	84	61.85^a,b^	32.32	99

The table shows values for control seeds and for seeds after passing through the digestive tract of each disperser.

a,b Values with different lowercase letters are significantly different in seed coat thickness for paired comparisons between treatments, after applying the Bonferroni correction factor (α’ = 0.016).

### Seed Germination

**Figure 1:**
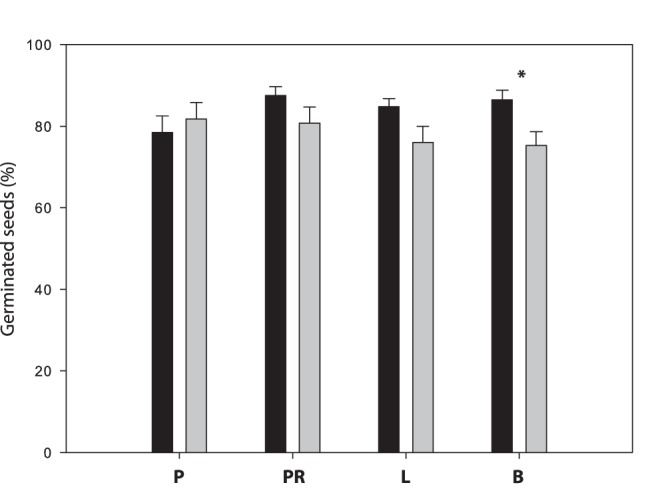
Average percentage and standard error of germinated seeds in *Rubia peregrina* (black bars) and *R.* *fruticosa* (grey bars) for different treatments: control with pulp (P), control with pulp removed (PR), lizards (L) and birds (B). Asterisk at the top of bars indicates significant difference between species across treatments after applying Bonferroni’s correction factor (α’ = 0.0018).

**Figure 2:**
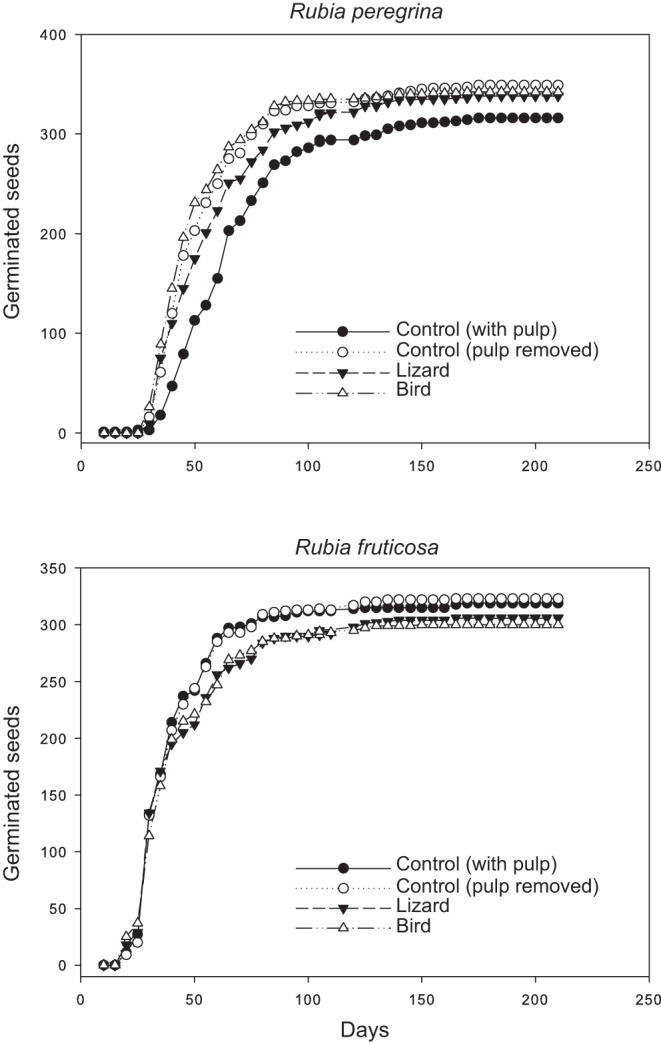
Seed germination rate of *Rubia peregrina* and *R.* *fruticosa* after the different treatments (seed dispersers and controls).

Total percentage germination was high in both species (90% and 83% for *R. peregrina* and *R. fruticosa*, respectively), which suggests an absence of seed dormancy. Seed germination ratio was significantly different between the *Rubia* species, but not between treatments (Table 2). However, the interaction between these two factors was significant, implying that the treatment effect varied between the plant species. Control seeds sown with pulp were the only case in which *R. peregrina* had a lower germination ratio than *R. fruticosa* (Fig. 1). In the rest of the treatments, *R. peregrina* had a higher germination than *R. fruticosa*. The effect of both dispersers was more detrimental in *R. fruticosa* (Fig. 1); and this difference between plant species was significant in the case of seeds ingested by birds (*F*_1,38_ = 16.565; *P*<0.001). However, this difference was marginally not significant for seeds ingested by lizards (*F*_1,38_ = 6.867; *P* = 0.0018; with *α*’ = 0.0018 after applying Bonferroni’s correction factor). Seed germination rate (speed of germination) was similar between the controls of the two *Rubia* species (Fig. 2), and also between individual plants (Breslow test, χ^2^ = 0.035, *df* = 19, *P* = 1.0) and treatments within each species (Breslow test, χ^2^ = 0.00, *df* = 3, *P* = 1.0).

**Table 2:** Results of GLM to test the effect of plant species (*Rubia peregrina* and *R. fruticosa*), treatment and mother plant on seed germination ratio.

Factor	Effect	*LR*	*d.f.*	*P*-value
Plant species	Fixed	19.406	1	<0.001
Treatment	Fixed	5.9841	3	0.1124
Plant species×Treatment	Fixed	17.183	3	<0.001
Mother plant	Random	65.345	19	<0.001

### Relationship between Seed Coat Thickness and Seed Germination Ratio

**Figure 3:**
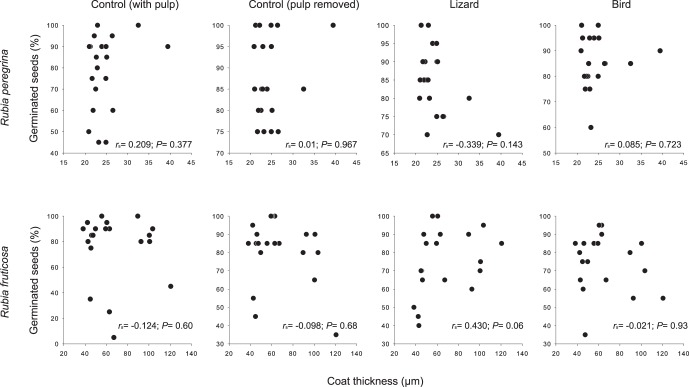
Relationship between average seed coat thickness and seed germination ratio of *Rubia fruticosa* and *R.* *fruticosa* across mother plants and within each treatment. Each dot corresponds to each mother plant (*n* = 20).

In *R. peregrina*, correlations between seed coat thickness and germination ratio were positive, although not significant, in all treatments except lizards (Fig. 3). Conversely, in *R. fruticosa*, correlations between seed coat thickness and seed germination were negative in all treatments, except for seeds ingested by lizards; in the latter case, the correlation was positive and marginally not significant (Fig. 3).

## Discussion

### Effect of Frugivore Treatment on Seed Coat Thickness

Seed coat thickness of *R. fruticosa* control seeds was nearly three times thicker than that of *R. peregrina* (Table 1). Wide variations in seed-coat scarification caused by differences in gut treatments in frugivorous passerine birds have previously been reported for different Mediterranean genera, including *Rubus* and *Rubia* [53]. Moreover, our results show that the level of scarification is also highly variable in congeneric species. Seed coat reduction in *R. fruticosa* was almost twice greater for seeds ingested by lizards than by birds, which is concordant with results from a previous study [43]. Lizards show clearly different digestive traits compared to birds: mouth treatment with teeth, much longer gut passage times (*G. galloti*: 7.2±2.6 days [54]; *S. atricapilla*: 36.6±24.6 min [55]) and stronger enzymatic action [43]. Such differences between the two types of dispersers are seen in the treatment received by seeds, specifically in the thinning of the seed coat after gut passage.

*Gallotia* lizards have been present in the Canary Islands since approximately 17–20 million years ago [38]. Conversely, although the oldest lineages of passerine birds may have inhabited the Canaries, the most complete fossil record of such birds in Macaronesia belongs to the Upper Pleistocene and Holocene (≈from 2.6 to <0.01 million of years ago) [40] and references therein). Moreover, colonization by *Sylvia atricapilla*, one of the most frugivorous birds in the Canaries, is thought to have taken place between 4 and 13 thousand years ago [39]. Thus, *R. fruticosa* seeds could have been exposed to the effect of digestive treatment by lizards during millions of years, whereas such an exposure to digestive treatment by birds could have been happening only for thousands of years. Lizards are also more effective seed dispersers of *R. fruticosa* than birds (González-Castro et al. in prep) and are much more abundant than birds in the xeric habitats of the Canaries inhabited by *R. fruticosa*. It therefore follows that the almost three times thicker seed coat of *R. fruticosa* compared to *R. peregrina* might be the outcome of selective pressures long exerted by lizards. Such a possibility is supported by the positive correlation between seed coat thickness and germination ratio of seeds ingested by lizards (Fig. 3). Unfortunately, with the data available to date it is not possible to assess if seedlings emerging from lizard-ingested seeds tend to produce seeds with a thicker coat than seedlings coming from not ingested seeds.

The effect of ingestion by either type of disperser on *R. peregrina* seed coats was relatively mild, coinciding with data from previous experiments with this species [53]. This is perhaps due to the fact that continental populations of *R. peregrina* have interacted for a long time with mammals in the Mediterranean Basin, which can exert a greater thinning of the *Rubia* seed coat than lizards and birds, as demonstrated by [43]. Hence, mammals might have selected for a thin but resistant seed coat because of the conservative nature of seed traits, regardless the continental or island origin. *R. fruticosa,* by contrast has a softer seed coat than *R. peregrina*. The former has a thicker, but more porous seed-coat texture, which probably buffers the effect of lizards’ digestive fluids (due to the coat thickness) and allows diffusion of water (due to the coat porosity) for a rapid germination when rains come in autumn [43], what is beneficial in relatively xeric environments [56]–[58] like the lowlands of the Canary Islands. A more porous seed coat could also be highly effective to withstand the hot summer season in the Canaries, when temperatures may surpass 35–40°C, as it enhances air circulation surrounding the embryo. In this respect, seed coat evolution might involve a compromise between protection of the embryo from destructive digestive processes and ability of seeds to survive and germinate.

Although the selective pressures that digestive action by frugivores have exerted on the evolution of seed coats have not yet been fully identified, we know from previous studies that the digestive processing of fruits and seeds by frugivorous animals has influenced the evolution of fruit and seed traits to enhance seed dispersal and maximize seed germination [59], [60]. On the other hand, the climatic conditions could have had a stronger effect than the lizards’ digestive system, because the climate might have been more stable than interactions with lizards.

### Effect of Frugivore Treatment on Seed Germination

The most significant insight from this study is the lack of differences in the effect on seed germination caused by the native and non-native two-disperser guilds in the two study systems. This is despite other studies have demonstrated that different disperser guilds can produce different patterns of seed germination in both *Rubia* species [33.43]. In the case of *R. peregrina*, the endemic lizard *Podarcis lilfordi* significantly reduced the seed germination ratio when compared to the passerine *Turdus merula* [33]. However, a clear negative effect has been recorded in another disperser group (mammals). The disruptive interaction of introduced rodents and lagomorphs with the native fleshy-fruited plants consumed on oceanic islands (e.g. the Canaries), along with which these plants have not evolved, has been demonstrated in *R. fruticosa* as well as in other native plants (*Lycium intricatum*, Solanaceae; *Asparagus pastorianus*, Convallariaceae; *Plocama pendula*, Rubiaceae) [43], [61], [62].

The contrasting results between different studies with the same species reflect the difficulty in generalizing about the effect of frugivorous animals on seed germination, as this aspect of seed dispersal is highly context-dependent [63], and seed germination experiments are no exception [33]. However, an important difference between these studies with *Rubia* species and our study is that we consider the variation among individual mother-plant seed sources (Table 2) [see [64] and references therein] whilst those other studies do not.

Another interesting result is the contrasting effect of fruit pulp on seed germination of the two *Rubia* species. Pulp removal due to fruit processing by frugivorous animals appears to be an important step during the germination process in some species [65], since a release from germination inhibitors and high osmotic pressures can alter germination processes [51]. In *R. peregrina*, a higher percentage of depulped seeds germinated than seeds sown with pulp (Fig. 1), so the main role that dispersers might be removing fruit pulp to enhance seed germination. In *R. fruticosa*, by contrast, the pulp surrounding seeds showed to have no detrimental effect on seed germination. Therefore, the species-dependence of the effect of pulp on seed germination processes reported by [65] could operate not only on different species, but also in closely related congeneric plant species. Furthermore, the stronger enhancing effect of pulp removal in *R. peregrina* (which interacted with a wider diversity of dispersers) than in *R. fruticosa*, might indicate that *R. peregrina* may be better adapted to dispersal by animals than *R. fruticosa*. Another possibility to explain the observed data is that interaction between *R. fruticosa* and lizards could be relatively recent, as a consequence of a later arrival of *R. fruticosa* in the Canary Islands. However, a study on phylogenetic history and hence time of divergence of these two species would confirm this possibility.

On the other hand, the enhancing effect of pulp removal observed in *R. peregrina* could be also (but not exclusively) related to some phenolic compounds, secondary metabolites that are very common and widespread in many plants, and which produce a double effect: 1) resistance of plants to pathogens (eg. Phytophagous insects or fungus) and, 2) inhibition of seed germination [57], [66]. Therefore, the continental species origin (*R. peregrina*) could evolve under the pressure of more fruit pathogens, having developed a higher amount of phenolic substances, and showing therefore a higher inhibition of seed germination, than the oceanic island plant (*R. fruticosa*).

### Why can Seeds of *R. peregrina* Survive the Treatment through the Digestive Tract of Non-natural Dispersers?

The finding that seeds of *R. peregrina* can resist the passage through a disperser like the large lizard *G. galloti*, with whom these plants have not evolved, might be related to the past climatic conditions of the Mediterranean Basin, when this area had a more subtropical climate during the Tertiary [67], [68]. Indeed, the major evolutionary differentiation phase of the Rubieae tribe, to which the genus *Rubia* belongs, coincided with the colonization of warm and xeric subtropical areas during the Upper Tertiary (≈55–65 Myr) [69] by many tropical taxa. This period overlapped for at least c. 25 Myr [70] with the origin of the three European lizard genera (*Lacerta*, *Teira* and *Podarcis*), which took place during the Oligocene (≈25–35 Myr) [71]. Therefore, *Rubia* seeds in the Mediterranean Basin might well have interacted with lizards for a long period when birds were not still present, not only in the islands but also in mainland areas.

It is also interesting to note that passerine birds in the Sylviidae family have been present in the Mediterranean for the last 15 Myr [72]. Specifically in the Balearics, the presence of the small *Podarcis* lizard (similar to present-day sizes) has been recorded since at least the Lower Pliocene (≈6 Myr ago) [73]. Therefore, the coexistence of these different guilds of vertebrate dispersers in the Mediterranean Basin and the ancestral origin of these *Rubia* species would support the fact that dispersers have a negligible effect on the evolution of seed germination in these fleshy-fruited plants. Our results actually support previous arguments regarding the conservative traits of plants throughout time [12]–[14]. Thus, some of the current fruit and seed traits could simply reflect an ancient interaction. This would indeed help explaining why large lizards in the Canaries act as legitimate dispersers of the Mediterranean *R. peregrina*, whereas birds do so for *R. fruticosa*.

## Concluding Remarks

The main message from this study is that the relationship between endozoochorous plants and the guild of dispersers with whom they evolved is rather unspecific. Furthermore, we find no evidence to attribute adjustments to the reciprocal adaptations of animals and plants over a long time period in isolated conditions. These results, a step forward from those based on fruit traits [10], [12], seem to confirm the conclusion of those two studies that current dispersers have not exerted a strong enough selective pressure to induce changes either on fruit traits or on seed germination responses. However, our findings on seed coat thickness in *R. fruticosa* suggest that *Gallotia* lizards might have modulated the evolution of seed coat toward a thicker but softer structure than that of its congeneric *Rubia peregrina*. To confirm such a hypothesis, more experiments are needed to test the heritability of seed coat thickness and also the relative importance of environmental conditions and lizard digestive treatment in determining seed coat evolution.

Lastly, it must be noted that despite the lack of adjustment between seed germination patterns in plant species and their native guild of dispersers, the latter might still have played an important role in other variables like fruit traits (e.g. color; *R. fruticosa* produces translucid fruits, in contrast to the black fruits of *R. peregrina*) or phenology (*R. fruticosa* fruits between March and May -coinciding partially with the period when lizards play their most important role as frugivores and seed dispersers [37]-, whereas *R. peregrina* fruits during autumn -with the migratory passerine pass-.
